# New Casbane and Cembrane Diterpenoids from an Okinawan Soft Coral, *Lobophytum* sp.

**DOI:** 10.3390/molecules21050679

**Published:** 2016-05-23

**Authors:** Prodip K. Roy, Runa Ashimine, Haruna Miyazato, Junsei Taira, Katsuhiro Ueda

**Affiliations:** 1 Department of Chemistry, Biology, and Marine Science, University of the Ryukyus, 1 Senbaru, Nishihara-cho, Okinawa 903-2013, Japan; 79earth.rararalune@gmail.com; 2 Department of Bioresources Engineering, Okinawa National College of Technology, 905 Henoko, Nago-shi, Okinawa 905-2192, Japan; br111436@edu.okinawa-ct.ac.jp (H.M.); taira@okinawa-ct.ac.jp (J.T.)

**Keywords:** casbane, cembrane, antibacterial, cytotoxicity, HCT 116 cells, anti-inflammatory

## Abstract

A new rare casbane-type diterpenoid **1** and two new cembrane diterpenoids **2**, **3** were isolated from an Okinawan soft coral, *Lobophytum* sp., together with four known cembrane diterpenoids **4**–**7**. Their structures were elucidated by extensive analysis of spectroscopic data (1D and 2D NMR, IR, and MS) and a molecular modeling study. The new isolates showed weak anti-bacterial activity, mild cytotoxicity against HCT116 cells, and anti-inflammatory effect in LPS/IFN-γ-stimulated RAW 264.7 macrophage cells.

## 1. Introduction

**Figure 1:**
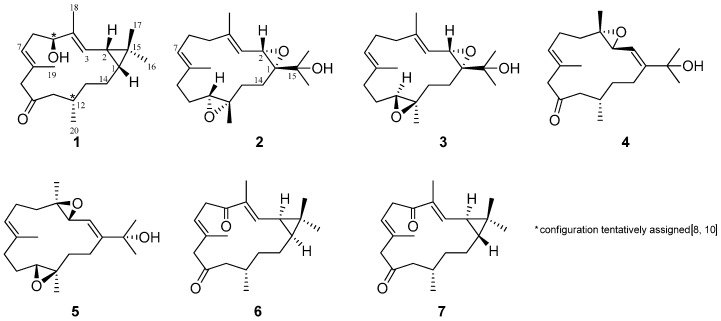
Chemical structures of compounds **1**–**7**.

Marine organisms are the amazing source of secondary metabolites due to the biodiversity of the oceans. The genus *Lobophytum* soft coral [1] is a good source for various kinds of secondary metabolites that have unique structures, promising bioactivity and it is also well-known for producing macrocyclic diterpenoids belonging to a large group of cembrane-type metabolites [2]. In our continuing research focused on the isolation and structure elucidation of bioactive secondary metabolites from Okinawan marine organisms [3,4,5,6,7], we examined a soft coral, *Lobophytum* sp., subsequently isolating a novel casbane **1** and two new cembranes **2**, **3**, along with four known cembrane diterpenoids **4**–**7** (Figure 1) [8,9,10,11]. Casbane-type diterpenoids are rare in Nature, the first being isolated from an enzymatic preparation of castor bean seedlings [12]; these compounds are also found in soft coral [10]. Most of the casbane-type diterpenoids are two ring-based macrocyclic structures where the junction of the two rings is *cis*-fused [10,13,14] and few molecules showed *trans* junctions [15]. These types of metabolites are of considerable pharmacological interest due to their unique structures and exhibit potential bioactivities, including cytotoxicity [16,17,18,19,20,21,22,23], anti-viral [23], anti-inflammatory [24,25,26] and antimicrobial activities [24]; casbane diterpenoids also display anti-proliferative activity [10]. Herein, we report the isolation, structure elucidation, and cytotoxicity of these new metabolites.

## 2. Results and Discussion

The Okinawan soft coral, *Lobophytum* sp. was collected from Irabu Island, Okinawa, and extracted with acetone. The acetone extract was partitioned between ethyl acetate and water. The ethyl acetate portion inhibited the growth of the Gram-positive bacterium *Staphylococcus aureus* and Gram-negative bacterium *Escherichia coli* with inhibition zones at 18 and 15 mm at 50 µg/disc, respectively. Repeated chromatographic and HPLC purification of the active crude extract resulted in the isolation of three new metabolites **1** (0.0023%, wet weight), **2** (0.0014%) and **3** (0.0005%) and four known metabolites **4** (0.0039%), **5** (0.0102%) **6** (0.0072%) and **7**, 0.0026%) identified by comparison of their NMR data with reported values [8,9,10,11].

**Figure 2:**
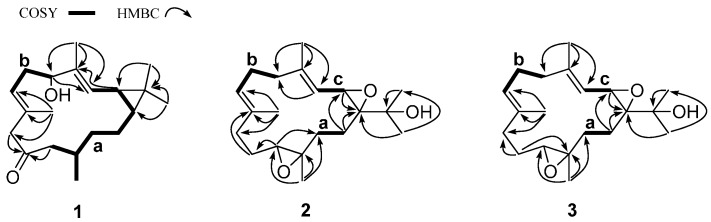
Partial structures of **1**–**3** based on COSY (bold line) and key HMBC correlations (arrow).

The molecular formula of **1** was determined to be C_20_H_32_O_2_ by high-resolution nanospray-ionization MS (HRNSIMS) (*m/z* 305.2470 [M + H]^+^, calcd. for C_20_H_33_O_2_, 305.2475), with five degrees of unsaturation. The IR spectrum showed hydroxyl and carbonyl groups (absorption bands at 3279 and 1701 cm^−1^). ^1^H- and ^13^C-NMR data (Table 1, Supplementary Material) suggested it was a diterpenoid and indicated the presence of a ketone (δ_C_ 210.6), two trisubstituted double bonds (δ_C_ 126.0 (δ_H_ 5.09 d, *J* = 9.5 Hz); 137.1; 124.1 (δ_H_ 4.90 t, *J* = 6.9 Hz); 131.3), one oxygenated carbon atom (δ_C_ 79.2 (δ_H_ 4.09 dd, *J* = 4.4, 11.0 Hz)), three sp^3^ methines (δ_C_ 31.4 (δ_H_ 0.65 ddd, *J* = 3.1, 9.0, 11.2 Hz), 25.3 (δ_H_ 1.22 dd, *J* = 9.5, 9.0 Hz), 31.6 (δ_H_ 1.88 m)), five sp^3^ methylenes (δ_C_ 33.0 (δ_H_ 2.34 m, 2.44 m); 51.9 (δ_H_ 3.15 d, *J* = 14.7 Hz and 2.82 d, *J* = 14.7 Hz); 52.4 (δ_H_ 2.22 d, *J* = 7.0 Hz); 37.2 (δ_H_ 1.15 m); 23.8 (δ_H_ 1.59 m, 0.75 m)) and five methyls (δ_C_ 15.7 (δ_H_ 1.01 s); 29.1 (δ_H_ 1.05 s); 10.3 (δ_H_ 1.64 s); 17.8 (δ_H_ 1.74 s) and 20.4 (δ_H_ 0.91 d, *J* = 6.6 Hz)). On the basis of ^1^H-^1^H COSY correlations, the two major spin systems (**a**: −CH_2_(11) −CH(12) −CH_3_(20) −CH_2_(13) −CH_2_(14) −CH(1) −CH_2_(2) −CH_2_(3) and **b**: −CH(5) −CH_2_(6) −CH(7)) were established (Figure 2).

Since compound **1** has three π-bonds, **1** must be bicyclic to satisfy the five degrees of unsaturation requirement. For the four singlet methyls, two were assigned to each of vinyl methyls in the two trisubstituted double bonds, based on heteronuclear multiple bond connectivity (HMBC) correlations (H_3_-18/C-3, -4, -5; H_3_-19/C-7, -8, -9) and the NMR chemical shifts; the remaining two were part of a *gem*-dimethyl group as indicated by HMBC correlations of H_3_-16/C-1, -15 and H_3_-17/C-2, -15 and COSY correlation between two cyclopropyl protons (δ_H_ 0.65 (ddd, *J* = 3.1, 9.0, 11.2 Hz), δ_H_ 1.22 (dd, *J* = 9.5, 9.0 Hz)), that indicated a tetrasubstituted cyclopropane ring in molecule **1**. An isolated methylene was associated with the ketonic carbonyl and a vinyl methyl (HMBC correlations of H_2_-9/C-10, -19), situated between C-8 and C-10. In addition, the tetrasubstituted cyclopropane ring associated with partial structure **a** was shown by HMBC correlations of H_3_-16/C-1, -15; H_3_-17/C-1, -2, -15 and H-2/C-4, -15 (Figure 2).

At this point in the structure determination, the partial structures (**a** with a cyclopropane ring, **b**, C-4–C-18, C-8–C-19–C-9–C-10 and C-15–C-16–C-17) were identified, but not assembled (Figure 2). HMBC correlations (H_3_-18/C-3, -4, -5; H_3_-19/C-7, -8, -9; H_2_-9, -11/C-10) finally connected these partial structures to give the 14-membered macrocyclic planar structure as a rare casbane-type diterpenoid (Figure 2). The two double bonds at C-3 and C-7, were assigned as *E* geometry due to the δ_C_ values of CH_3_-18 and CH_3_-19 (<20 ppm) [27]. The junction of the two rings at carbons C-1/C-2 was suggested to be *cis* orientation by comparison of the ^13^C chemical shifts of the geminal methyls [δ_C_ 15.7 (C-16) and 29.1 (C-17)] in **1** with those of the known *cis*-fused casbane diterpenes [10,13,14]. The coupling constant (*J* = 9.0 Hz) between H-1 (δ_H_ 0.65 (ddd, 3.1, 9.0, 11.2 Hz)) and H-2 ((δ_H_ 1.22 (dd, *J* = 9.5, 9.0 Hz)) and an NOE of H-1/H-2 also supported *cis* configuration of the cyclopropane protons.

**Figure 3:**
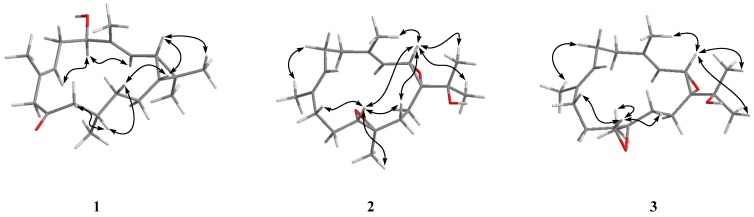
Computer-generated model of **1**–**3** using MM2 force calculations and key NOE correlations.

The relative stereo structure of **1** was tentatively assigned by 1D Nuclear Overhauser Effect (NOE) experiments (Figure 3) and by comparison of the NMR data for **1** with those reported for congeners of **1** [10,13,14]. In the NOE experiments of **1**, irradiation of the H-1 signal revealed NOEs with H-2, and irradiation of the H-2 signal showed NOEs with H-1, H_3_-17, suggesting these protons were on the same face (Figure 3). The NOEs between H-1 and H-13 and H-12/H-11, -13, suggested that H_3_-20 was on the side opposite these protons in the molecule. Irradiation of the H-5 signal revealed NOEs with H-3, -7 but not with H-2 and irradiation of the H-3 signal, showed an NOE with H-5 but not H-2. So, H-5 and H-2 could be opposite sides of the molecule. Unfortunately, attempts to prepare MTPA esters for determination of the absolute stereochemistry failed because of its instability and the small quantity of compound **1** available.

The HRNSIMS (*m*/*z* 321.2418 [M + H]^+^, calcd. for C_20_H_33_O_3_, 321.2424) of **2** suggested the molecular formula C_20_H_32_O_3_, which accounted for five degrees of unsaturation. The IR spectrum showed hydroxyl and epoxide functionalities (absorption bands at 3481, 1295 and 1252 cm^−1^). ^1^H- and ^13^C-NMR data (Table 1), coupled with the molecular formula C_20_H_32_O_3_, suggested it was a diterpenoid derivative and indicated the presence of one oxygenated carbon atom (δ_C_ 70.1), two epoxides (δ_C_ 57.5 (δ_H_ 3.75 d, *J* = 4.2 Hz); δ_C_ 68.2 and δ_C_ 61.6 (δ_H_ 2.71 dd, *J* = 3.4, 9.1 Hz); δ_C_ 61.2), two trisubstituted double bonds (δ_C_ 118.8 (δ_H_ 5.08 brd, *J* = 4.2 Hz); δ_C_ 141.9 and δ_C_ 126.2 (δ_H_ 5.15 t, *J* = 6.1 Hz); δ_C_ 133.7), six sp^3^ methylenes (δ_C_ 38.9 (δ_H_ 2.27 m); 24.5 (δ_H_ 2.24 m); 36.8 (δ_H_ 2.23 m, 2.04 m); 24.3 (δ_H_ 2.25 m, 1.96 m); 35.2 (δ_H_ 1.99 m); 25.4 (δ_H_ 1.86 m, 1.34 m)) and five methyls (δ_C_ 26.2 (δ_H_ 1.25 s); 26.7 (δ_H_ 1.31 s); 17.7 (δ_H_ 1.70 s); 15.3 (δ_H_ 1.64 s) and 17.0 (δ_H_ 1.24 s)). Since compound **2** has two π-bonds and two epoxides, **2** must be monocarbocyclic to fulfill the five degrees of unsaturation requirement. Three major spin system (**a**: −CH_2_(13)−CH(14), **b**: −CH(5)−CH(7), and **c**: −CH(2) −CH(3)), were identified from the ^1^H-^1^H COSY correlations (Figure 2). The two epoxides were trisubstituted, based on HMBC correlations (H-2/C-1, H_2_-14/C-1, -2). For the five methyls, two were assigned to each of vinyl methyls in the two trisubstituted double bonds, based on HMBC correlations (H_3_-18/C-3, -4, -5 and H_3_-19/C-7, -8, -9) and the NMR chemical shifts; one was associated with an epoxide at C-12 (HMBC correlations of H_3_-20/C-11, -12, -13), and the remaining two were part of a gem-dimethyl group, and were associated with another epoxide at C-1 as indicated by HMBC correlations of H_3_-16/C-1, -15, -17 and H_3_-17/C-15, -16. On the basis of HMBC correlations (Figure 2), three partial structures (**a**, **b** and **c**) and other fragments could be connected to give the planar structure **2** as a cembrane-type diterpenoid (Figure 2).

The relative configuration of **2** was assigned by detailed analysis of 1D NOE experiments. NOE correlations between H-2/H-11, H-2/H-13, H-2/H_3_-16, H-2/H_3_-17, H-2/H_3_-18, H-11/H-9, H-11/H-13 and H-11/ H_3_-20 implied that these protons were on the same face (Figure 3). The NOE correlations between H-2/H_3_-18 and H-6/H_3_-19, and δ_C_ values of CH_3_-18 and CH_3_-19 (<20 ppm) [27] suggested that the two double bonds at C-3 and C-7 should be assigned as *E* geometry.

The molecular formula of **3** (C_20_H_32_O_3_) was the same as **2**, as inferred by HRNSIMS (*m/z* 321.2419 [M + H]^+^, calcd. for C_20_H_33_O_3_, 321.2424). The ^1^H- and ^13^C-NMR spectra (Table 1) of **3** were very similar to those of **2**. Extensive analysis of 1D and 2D NMR data, and comparison of the NMR data with those of **2** led to the same planar structure as that of **2**. Since the NOEs observed for the portions at C1, C2, C3, C4, C7 and C8 in **3** resembled those described above for **2**, both compounds possess identical stereochemistry in these portions. An NOE between H-11/H_3_-20 in **3**, along with no NOE effect on H-2 and H-11 upon irradiation of H-11, suggested that the protons H-11 and H_3_-20 in **3** were on the same face (the opposite of that found in **2**) (Figure 3). Therefore, compounds **3** and **2** were epoxide moiety stereoisomers at C-12.

**Figure 4:**
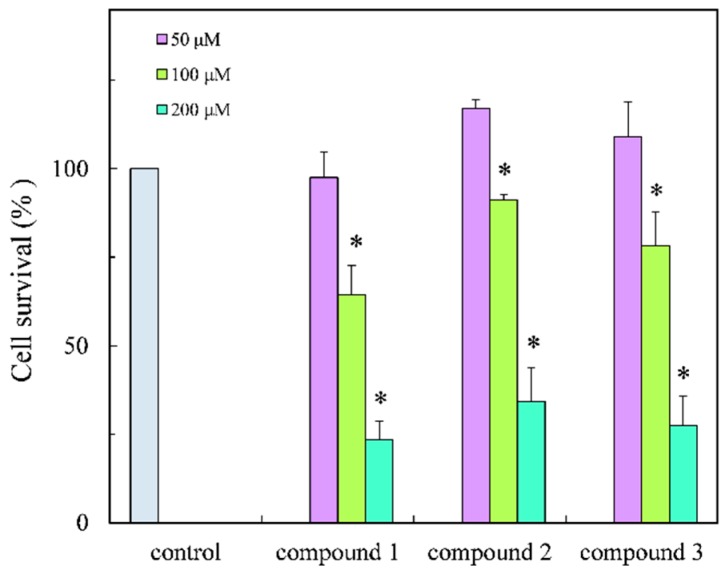
Cytotoxicity of **1**–**3** against HCI116 colon cancer cells. Significance * *p* < 0.01 was considered statistically significant for control.

**Figure 5:**
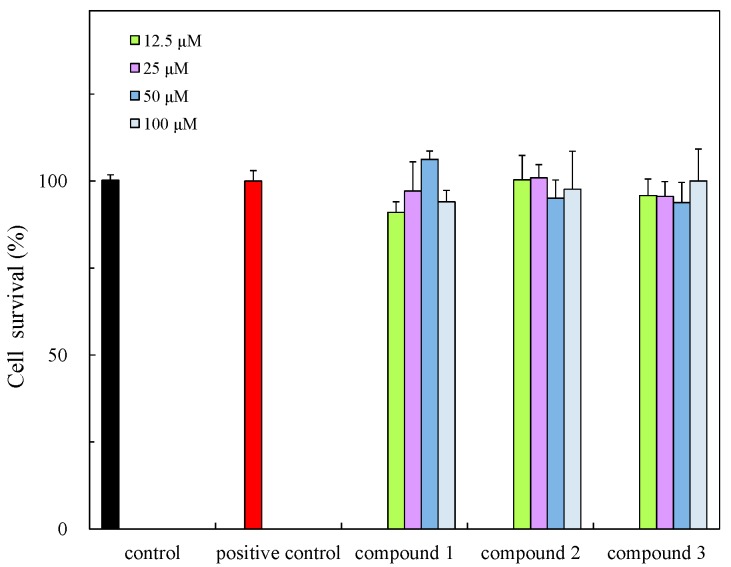
Cytotoxicity of **1**–**3** for NO production in LPS/IFN-γ stimulated RAW 264.7 macrophage cells.

**Figure 6:**
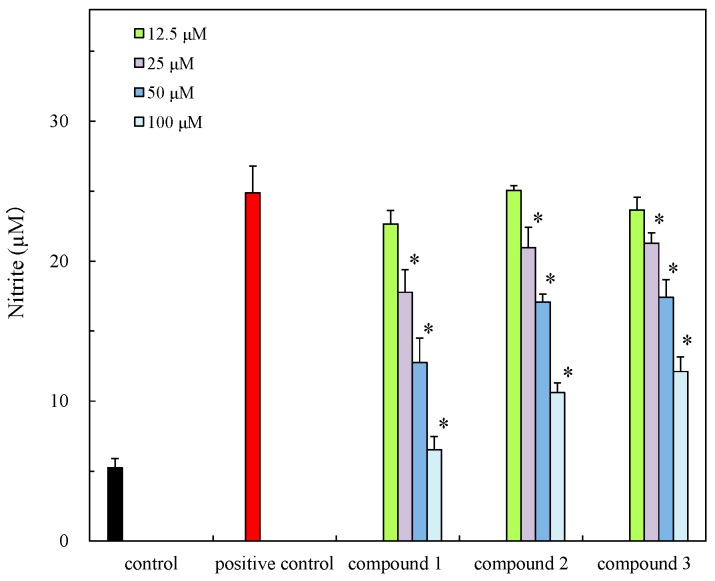
Anti-inflammatory effect of **1**–**3** against NO production in LPS-stimulated RAW 264.7 macrophage cells. Significance * *p* < 0.01 was considered statistically significant for positive control.

The isolates were evaluated for antibacterial activity using the paper disc method [28] against *S. aureus*, *S. enterica* and *E. coli* and new isolates also evaluated for cytotoxicity and anti-inflammatory effect in cells (Table 2). The isolates showed weak anti-bacterial activity and new compounds exhibited cytotoxicity against HCT 116 cells (Figure 4) but this was weaker than those of previously reported compounds, for example alcyonolide and its congeners isolated from soft coral *Cespitularia* sp. were in the IC_50_ 5.85–91.4 µM range [4]. The anti-inflammatory activity of compounds **1**–**3** was also evaluated in LPS/IFN-γ-stimulated RAW 264.7 macrophage cells under non-cytotoxic concentration ranges (Figure 5 and Figure 6). The compounds suppressed NO production in a dose dependent manner, indicating the compounds have the anti-inflammatory effect. The inhibition was similar to that of flavonoids, but they were low levels (IC_50_ (µM), 41.2–74.8) by comparison with alcyonolide congeners (2–8 µM) [29] and marine carotenoids (6.25–25 µM), such as fucoxanthin and fucoxanthinol [30].

## 3. Experimental Section

### 3.1. General Procedures

Optical rotation was measured using a JASCO P-1010 polarimeter (JASCO International Co. Ltd., Tokyo, Japan). Nuclear magnetic resonance (NMR) spectra were recorded on an Avance III 500 spectrometer (Bruker, Rheinstetten, Germany) in CDCl_3_. Chemical shifts and coupling constants were given as δ and Hz, respectively and ^1^H- and ^13^C- chemical shifts were referenced to the solvent peaks (δ_H_ = 7.26 and δ_C_ = 77.24). Infrared (IR) spectra were recorded on a JASCO FT/IR-6100 Fourier Transform Infrared Spectrometer (JASCO International Co. Ltd.). High-resolution mass spectra (HRMS) were obtained on an LTQ Orbitrap hybrid mass spectrometer (Thermo Scientific, Bemen, Germany) equipped with a nanospray ionization (NSI) source. Open column chromatography was performed on Kieselgel 60 (70–230 mesh, Merck, Darmstadt, Germany). High performance liquid chromatography (HPLC) was performed using a COSMOSIL Si60 HPLC column (5 SL, Φ 10 × 250 mm, Nacalai tesque Inc, Osaka, Japan). Analytical thin layer chromatography (TLC) was performed using Kieselgel 60 F_254_ DC-fertigplatten (Merck). All solvents were reagent grade.

### 3.2. Animal Materials

The soft coral *Lobophytum* sp. (220.0 g, wet weight) was collected by hand during low tide from the coast of Irabu Island, Okinawa, Japan, in March 2013, and identified as a *Lobophytum* sp. A voucher specimen was deposited at University of the Ryukyus (Specimen No. 13033102).

### 3.3. Extraction and Isolation

The soft coral was transported to the lab and extracted with acetone (2 L × 3). After filtration, extracts were concentrated under reduced pressure to form an acetone extract. The acetone extract was partitioned between H_2_O (200 mL) and EtOAc (200 mL × 2). The EtOAc part was evaporated *in vacuo* to give a crude extract (2.41 g) that inhibited the growth of the Gram-positive bacterium *Staphylococcus aureus* and Gram-negative bacterium *Escherichia coli* with inhibition zones of 18 and 15 mm, respectively, at 50 µg/disc. The active crude extract was first chromatographed over silica gel to give 19 fractions (hexane/EtOAc/MeOH gradient). On the basis of its ^1^H-NMR spectrum, fraction 8 was subjected to further purification by HPLC. An aliquot (102.3 mg) of fraction 8 (213.4 mg) was purified by HPLC (a COSMOSIL Si-60 column SiO_2_) using hexane/EtOAc (7:3) to afford new diterpenoids **1** (2.5 mg), **2** (1.5 mg), **3** (0.6 mg) and known diterpenoids **4** (4.2 mg) and **5** (10.8 mg). An aliquot (44.2 mg) of fraction 5 (130.4 mg) was purified by HPLC using hexane/EtOAc (4:1) to afford known diterpenoids **6** (5.4 mg) and **7** (2.0 mg).

Compound **1**: Colorless oil; ${\left[\alpha \right]}_{D}^{31.4}$ −111.4 (*c* 0.07 CH_3_OH); FT/IR *ν*_max_ (film) 3279, 2921 and 1701 cm^−1^; ^1^H-NMR and ^13^C-NMR data are listed in Table 1; HRNSIMS *m/z* 305.2470 [M + H]^+^ (calcd. for C_20_H_33_O_2_, 305.2475).

Compound **2**: Colorless oil; ${\left[\alpha \right]}_{D}^{31.5}$ +12.0 (*c* 0.05 CH_3_OH); FT/IR *ν*_max_ (film) 3481, 2932, 1295 and 1252 cm^−1^; ^1^H and ^13^C-NMR (CDCl_3_) data are listed in Table 1; HRNSIMS *m/z* 321.2418 [M + H]^+^ (calcd. for C_20_H_33_O_3_, 321.2424).

Compound **3**: Colorless oil; ${\left[\alpha \right]}_{D}^{31.7}$ −16.6 (*c* 0.06 CH_3_OH); FT/IR *ν*_max_ (film) 3465, 2930, 1254 and 1166 cm^−1^; ^1^H and ^13^C-NMR (CDCl_3_) data are listed in Table 1; HRNSIMS *m/z* 321.2419 [M + H]^+^ (calcd. for C_20_H_33_O_3_, 321.2424).

### 3.4. Molecular Mechanics Calculations

Implementation of the MM2 force field [32] in ChemBioOffice Ultra 12.0 software (Cambridge Soft Corporation, Cambridge, MA, USA) was used to calculate molecular models.

### 3.5. Anti-Bacterial Assay

The paper disk diffusion method [28] was used to evaluate the anti-bacterial activity of compounds **1**–**5**, using the bacterial strains *Staphylococcus aureus*, *Salmonella enterica* and *Escherichia coli.* The strains were received from the Biological Resource Center (NBRC, Tokyo, Japan), Japan and cultured in an agar medium containing polypeptone (10 g/L distilled water), yeast (2 g/L distilled), MgSO_4_·7H_2_O (1 g/L distilled) and agar (15 g/L distilled). The medium was autoclaved and transferred into petri dishes. The bacterial inoculum was evenly spread on the above agar medium. Each methanolic solution of the test compounds was perfused (25 µg/25 µL) to a sterilized disc (Φ 8 mm, Toyo Roshi Kaisha, Ltd., Tokyo, Japan). After the removal of the solvent, the disks containing test compounds were placed on seeded bacterial lawn on the agar surface. The plate was incubated for 2 days at 30 °C and then the inhibition zone sizes were measured.

### 3.6. Cell Culture

HCT116 human colon cancer cells (ATCC, Manassas, VA, USA) and RAW 264.7 cells (mouse macrophages, American Type Culture Collection) were cultured in DMEM (Gibco-BRL, Life Technologies, South San Francisco, CA, USA) medium (including 10% FBS, 100 U/mL penicillin and 100 μg/mL streptomycin) at 37 °C in a 5% CO_2_ atmosphere.

### 3.7. Cell Viability

The MTT assay was used to examine the cytotoxity of compounds **1**–**3**. Briefly, HCT116 cells were seeded at a density of 5.0 × 10^5^ cells/mL in 96-well plate and cultured for 24 h with or without the test compound. After the culture, MTT (0.05%) was added to each well and incubated for 2 h, and then suspension was removed. Extraction with DMSO (50 μL) was measured at 540 nm with the reference at 655 nm using a microplate reader (BIORAD model 550, BIO-RAD, Hercules, CA, USA).

### 3.8. Anti-Inflammatory Effect on Nitrite Production on RAW 264.7 Macrophages

The RAW 264.7 cells (2.5 × 10^6^ cells /mL) were treated with the compounds **1**–**3** in the presence of LPS (100 ng/mL), L-arginine (2 mM), and IFN-γ (100 U/mL) in 96-well microplate. Cells with or without LPS, IFN-γ and L-arginine were used as the positive control and the control, respectively. After culturing for 17 h, the nitrite concentrations in the medium were determined by previously reported method [31].

### 3.9. Statistical Analysis

Data were expressed as mean ± SD. Statistical significance (*p* < 0.01) was analyzed by Student’s *t*-tests.

## 4. Conclusions

Seven diterpenoids **1**–**7**, including three new compounds **1**–**3,** were isolated from the Okinawan soft coral, *Lobophytum* sp. Their relative stereostructures were established by spectroscopic analysis (NMR, IR, and MS) and comparisons with similar reported metabolites. The new isolates showed weak antibacterial activity, mild cytotoxicity against human colon cancer cells and showed anti-inflammatory effect in LPS/IFN-γ-stimulated RAW 264.7 macrophage cells.

## Figures and Tables

**Table 1:** ^1^H- (500 MHz) and ^13^C-(125 MHz) NMR data for **1**–**3** in CDCl_3_.

C No.	1		2		3	
	δ_H_ (mult., *J*/Hz)	δ_C_	δ_H_ (mult., *J*/Hz)	δ_C_	δ_H_ (mult., *J*/Hz)	δ_C_
1	0.65 (ddd, 3.1, 9.0, 11.2)	31.4 (CH)		68.2 (C)		68.0 (C)
2	1.22 (dd, 9.5, 9.0)	25.3 (CH)	3.75 (d, 4.2)	57.5 (CH)	3.80 (d, 5.8)	57.9 (CH)
3	5.09 (brd, 9.5)	126.0 (CH)	5.08 (brd, 4.2)	118.8 (CH)	4.79 (brd, 5.8)	121.0 (CH)
4		137.1 (C)		141.9 (C)		140.0 (C)
5	4.09 (dd, 4.4, 11.0)	79.2 (CH)	2.27 (m)	38.9 (CH_2_)	2.11 (m)	38.9 (CH_2_)
6	2.44 (m)	33.0 (CH_2_)	2.24 (m)	24.5 (CH_2_)	2.31 (m)	24.8 (CH_2_)
	2.34 (m)					
7	4.90 (dd, 6.9, 6.9)	124.1 (CH)	5.15 (t, 6.1)	126.2 (CH)	5.10 (t, 5.2)	125.8 (CH)
8		131.3 (C)		133.7 (C)		134.0 (C)
9	3.15 (d, 14.7)	51.9 (CH_2_)	2.23 (m)	36.8 (CH_2_)	2.18 (m)	36.8 (CH_2_)
	2.82 (d, 14.7)		2.04 (m)			
10		210.6 (C)	2.25 (m)	24.3 (CH_2_)	2.29 (m)	23.7 (CH_2_)
			1.96 (m)		2.02 (m)	
11	2.22 (d, 7.0)	52.4 (CH_2_)	2.71 (dd, 3.4, 9.1)	61.6 (CH)	2.59 (dd, 3.3, 10.6)	61.9 (CH)
12	1.88 (m)	31.6 (CH)		61.2 (C)		61.4 (C)
13	1.15 (m)	37.2 (CH_2_)	1.99 (m)	35.2 (CH_2_)	2.27 (m)	35.1 (CH_2_)
					1.96 (m)	
14	1.59 (m)	23.8 (CH_2_)	1.86 (m)	25.4 (CH_2_)	2.09 (m)	24.3 (CH_2_)
	0.75 (m)		1.34 (m)			
15		21.0 (C)		70.1 (C)		70.8 (C)
16	1.01 (s)	15.7 (CH_3_)	1.25 (s)	26.2 (CH_3_)	1.29(s)	25.4 (CH_3_)
17	1.05 (s)	29.1 (CH_3_)	1.31 (s)	26.7 (CH_3_)	1.28 (s)	26.5 (CH_3_)
18	1.64 (s)	10.3 (CH_3_)	1.70 (s)	17.7 (CH_3_)	1.70 (s)	17.0 (CH3)
19	1.74 (s)	17.8 (CH_3_)	1.64 (s)	15.3 (CH_3_)	1.64 (s)	14.9 (CH_3_)
20	0.91 (d, 6.6)	20.4 (CH_3_)	1.24 (s)	17.0 (CH_3_)	1.25 (s)	16.0 (CH_3_)

**Table 2:** Antibacterial activity, cytotoxicity and anti-inflammatory effect of compounds **1**–**5**.

Compound	Antibacterial Activity ^a^			Cytotoxicity (IC_50_, µM)	Anti-Inflammatory Effect (IC_50_, µM)
	*S. aureus*	*S. enterica*	*E. coli*	HCT116 cells	RAW 256.7 cells
**1**	10	N.A ^b^	10	135.57	41.21
**2**	9	12	10	177.11	64.96
**3**	9	10	10	153.11	74.76
**4**	10	N.A ^b^	12	N.T ^c^	N.T ^c^
**5**	10	N.A ^b^	15	N.T ^c^	N.T ^c^
Streptomycin sulfate	15	N.T ^c^	13	N.T ^c^	N.T ^c^

^a^ Inhibition zone in mm at 25 µg/disc, ^b^ Not active, and ^c^ Not tested [31].

## Supplementary Materials

Supplementary materials can be assessed at: http://www.mdpi.com/1420-3049/21/5/679/s1.
