# Supplementary material for: New Casbane and Cembrane Diterpenoids from an Okinawan Soft Coral, Lobophytum sp
Source: Molecules. 2016 May 23;21(5):679. doi: 10.3390/molecules21050679 (PMC6273427; doi:10.3390/molecules21050679)
Supplement: Supplementary file 1 [file molecules-21-00679-s001.pdf]

Supplementary Materials: New Casbane and Cembrane Diterpenoids from an Okinawan Soft Coral, *Lobophytum* sp.

Prodip K. Roy, Runa Ashimine, Haruna Miyazato, Junsei Taira and Katsuhiro Ueda

Contents

Figure S1. ^{1}H-NMR spectra (500 MHz) of **1**.

Figure S2. ^{13}C-NMR spectrum (125 MHz) of **1**.

Figure S3. HMQC spectra of **1**.

Figure S4. ^{1}H–^{1}H COSY spectrum of **1**.

Figure S5. HMBC spectrum of **1** in CDCl3.

Figure S6. HRNSIMS spectrum of **1**.

Figure S7. ^{1}H-NMR spectra (500 MHz) of **2**.

Figure S8. ^{13}C-NMR spectrum (125 MHz) of **2**.

Figure S9. HMQC spectra of **2**.

Figure S10. ^{1}H–^{1}H COSY spectrum of **2**.

Figure S11. HMBC spectrum of **2** in CDCl3.

Figure S12. HRNSIMS spectrum of **2**.

Figure S13. ^{1}H-NMR spectra (500 MHz) of **3**.

Figure S14. ^{13}C-NMR spectrum (125 MHz) of **3**.

Figure S15. HMQC spectra of **3**.

Figure S16. ^{1}H–^{1}H COSY spectrum of **3**.

Figure S17. HMBC spectrum of **3** in CDCl3.

Figure S18. HRNSIMS spectrum of **3**.

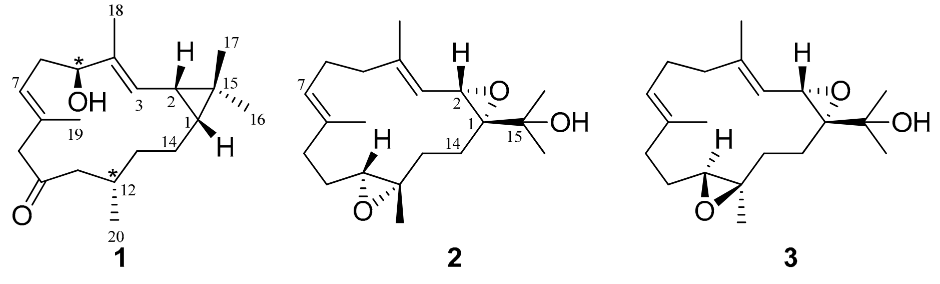

1 **2** **3**

Isolated compounds **1**–**3** from *Lobophytum* sp.

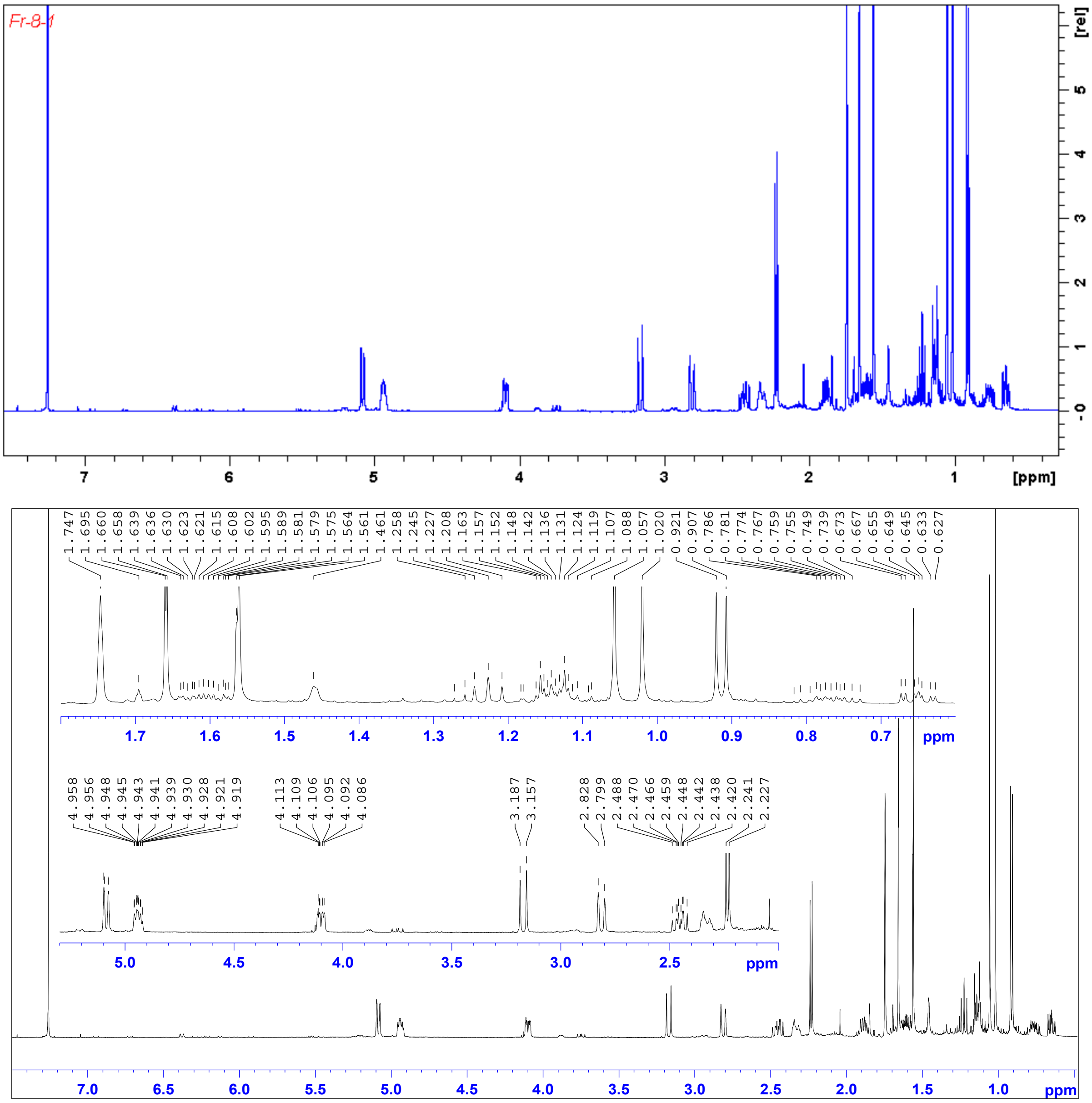

Figure S1. ^{1}H-NMR spectra (500 MHz) of **1**.

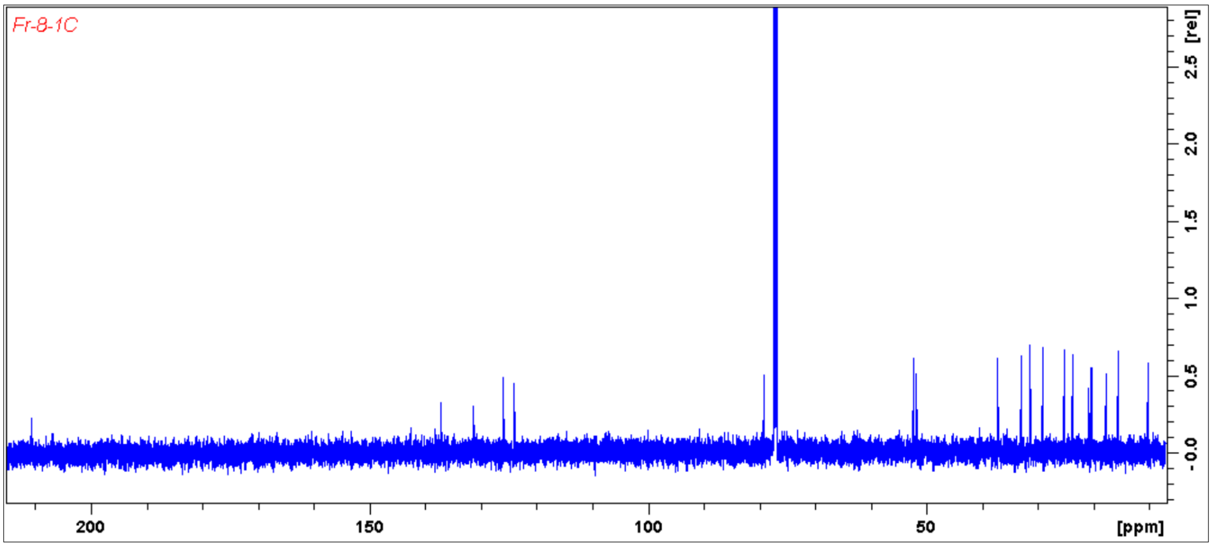

Figure S2. ^{13}C-NMR spectrum (125 MHz) of **1**.

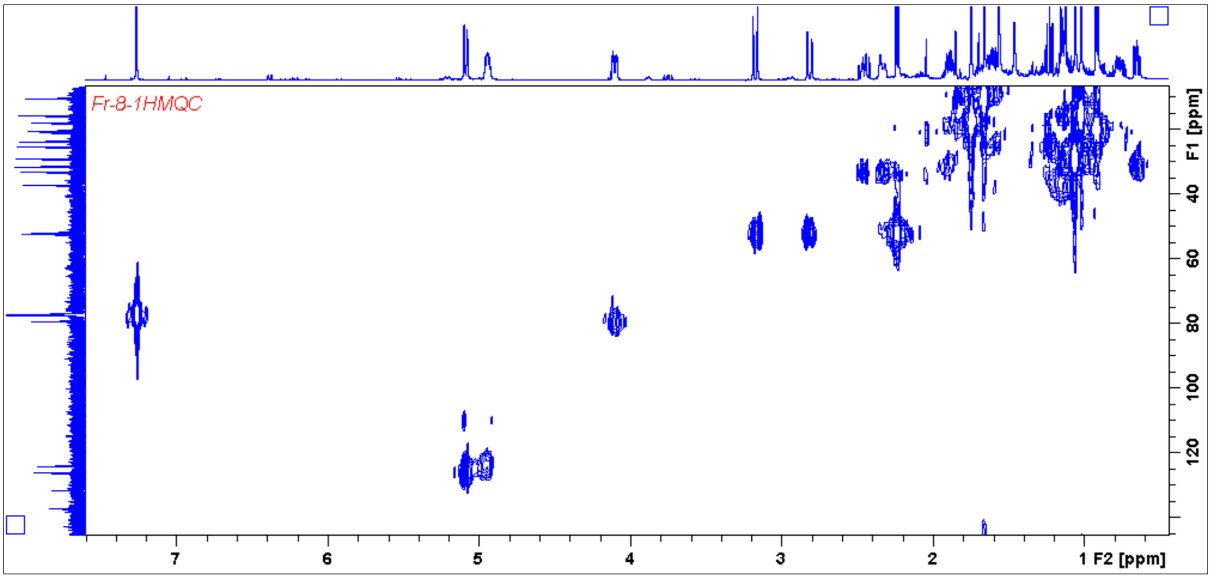

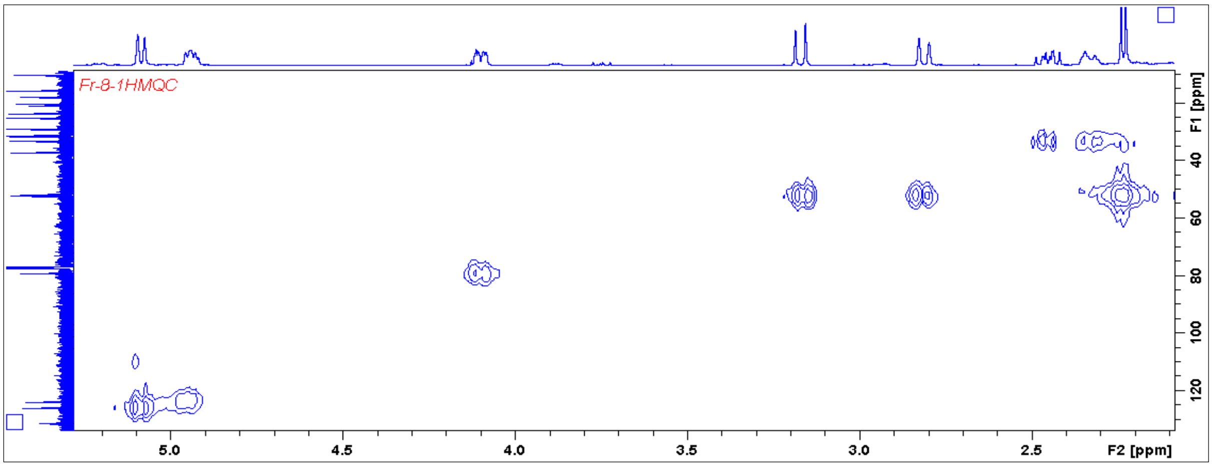

Figure S3. *Cont.*

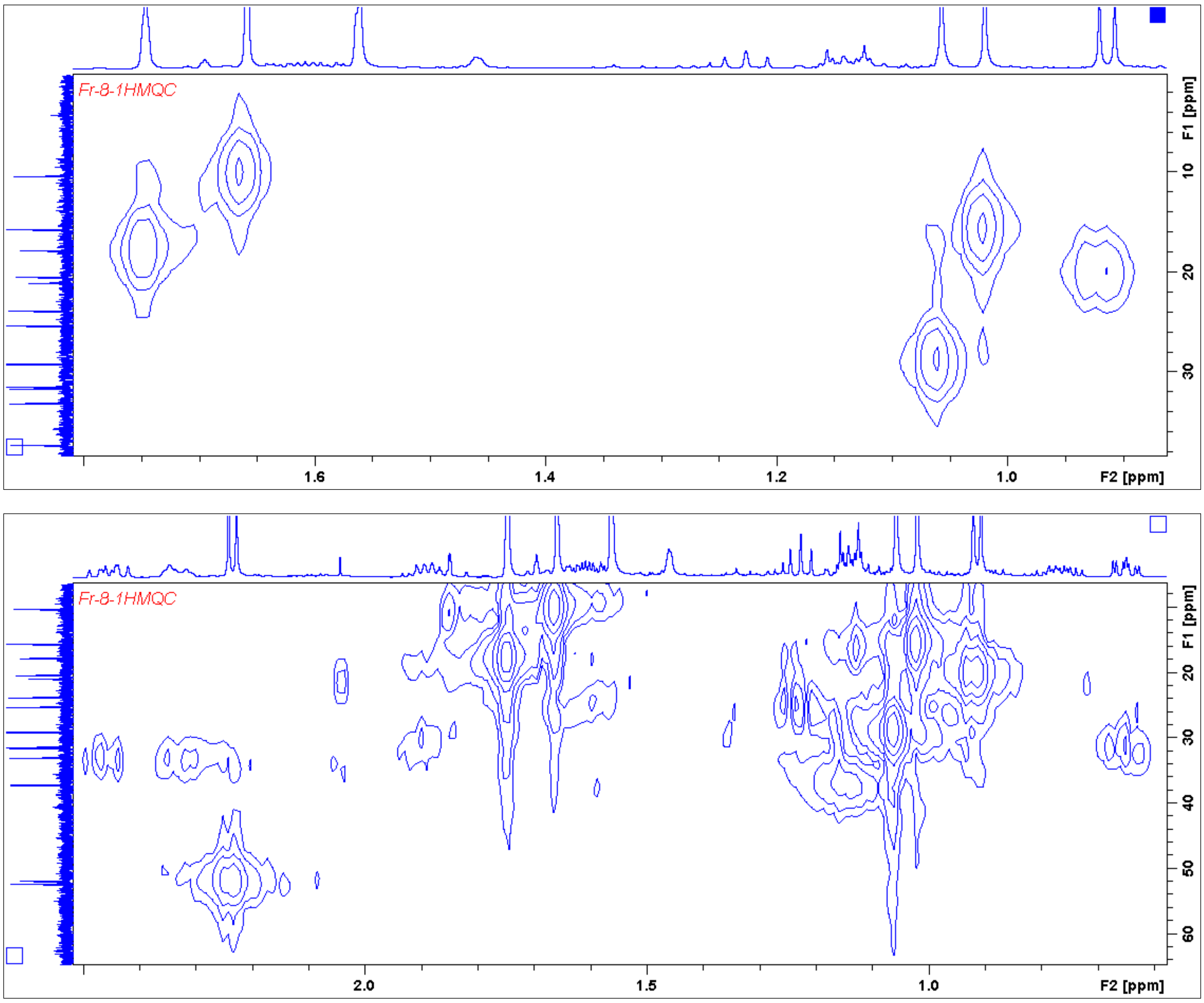

Figure S3. HMQC spectra of **1**.

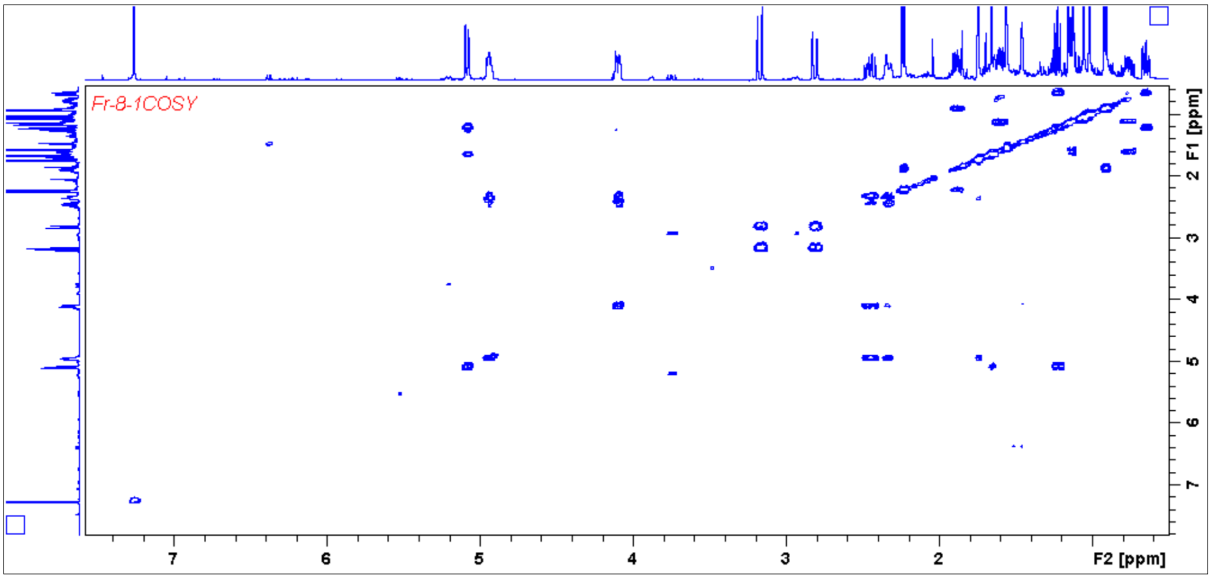

Figure S4. ^{1}H–^{1}H COSY spectrum of **1**.

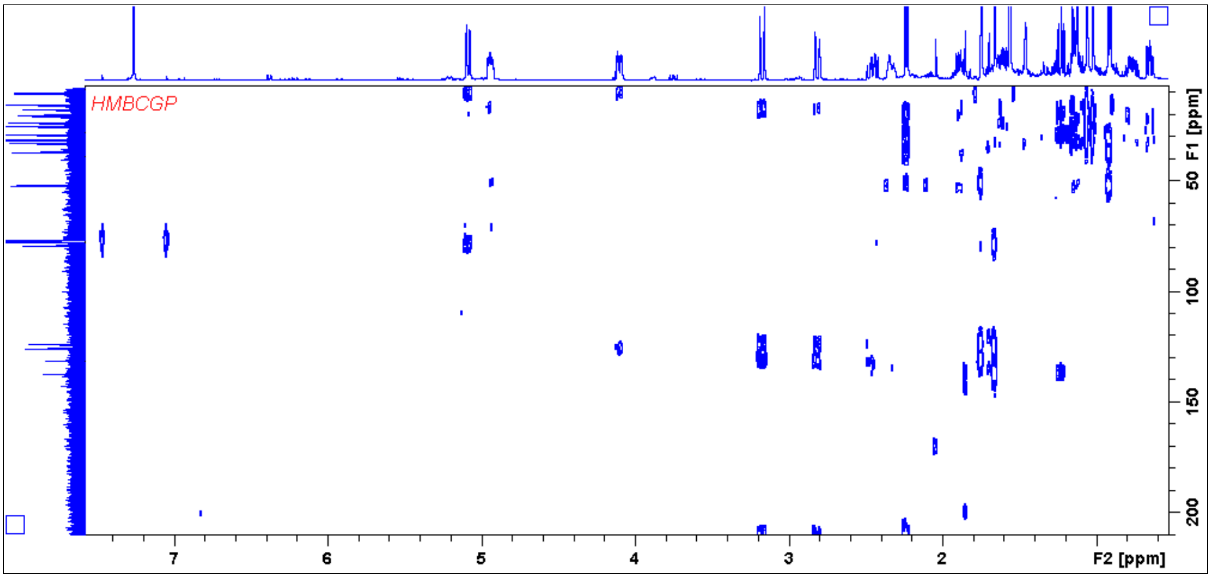

Figure S5. HMBC spectrum of **1** in CDCl3.

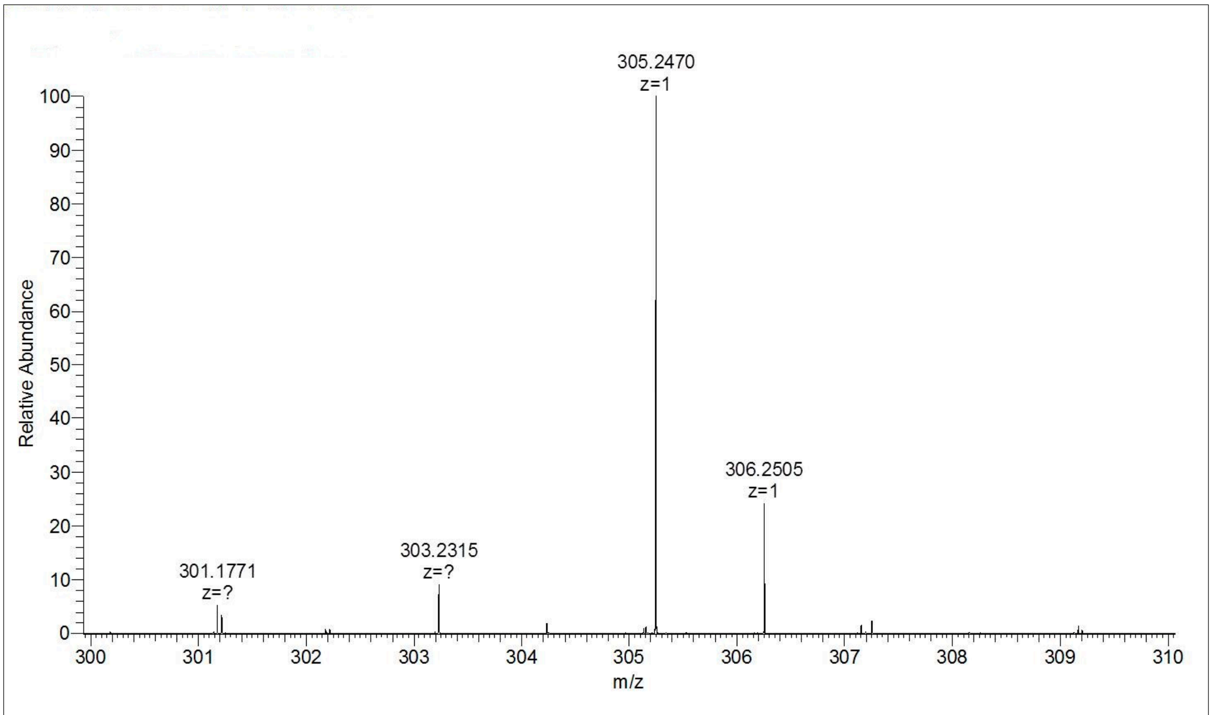

	m/z	Theo. Mass	Delta (mmu)	RDB equiv.	Composition
[M+H]+	305.2470	305.2475	-0.50	4.5	$C_{20}H_{33}O_2$

Figure S6. HRNSIMS spectrum of **1**.

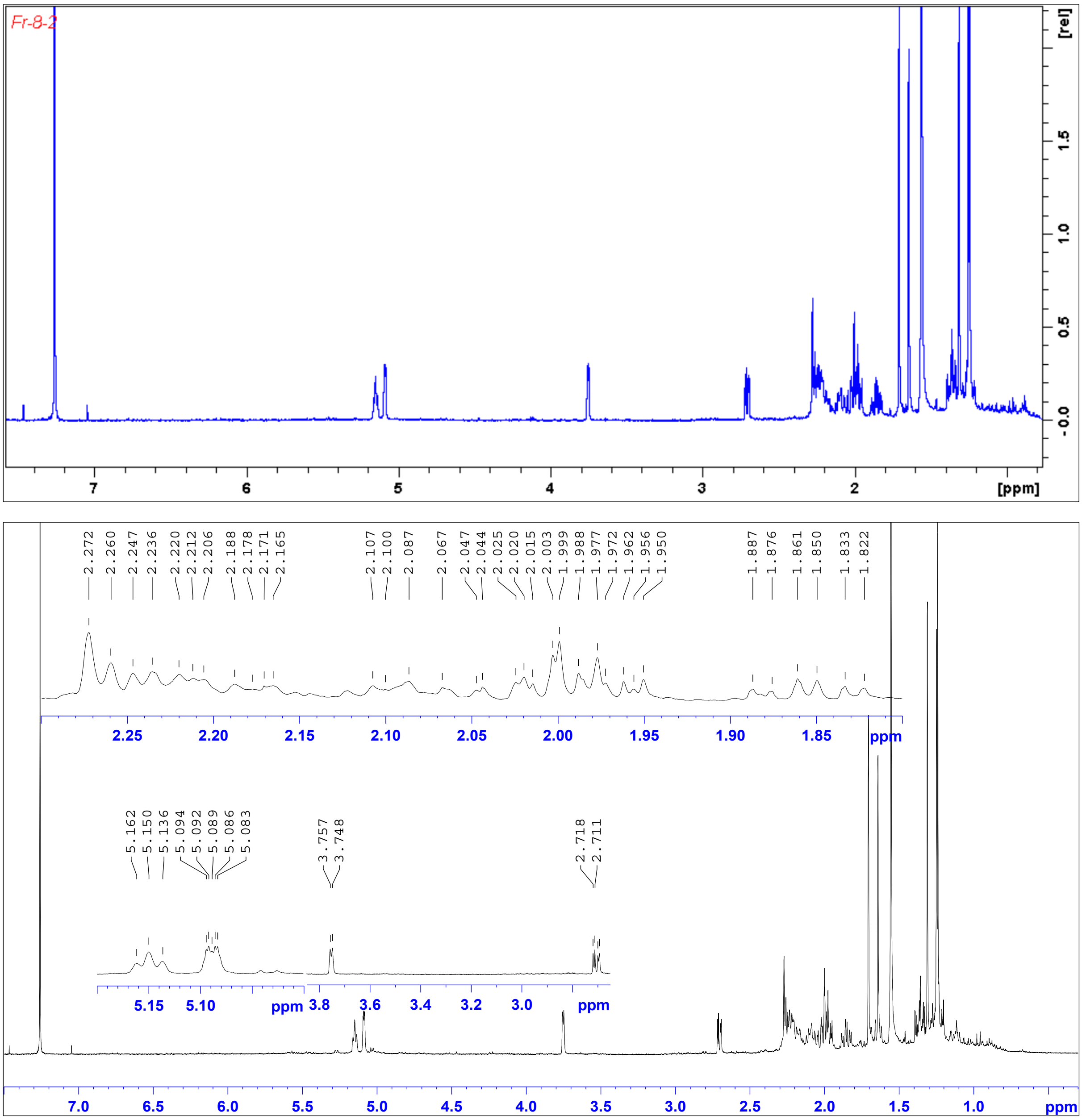

Figure S7. ^{1}H-NMR spectra (500 MHz) of **2**.

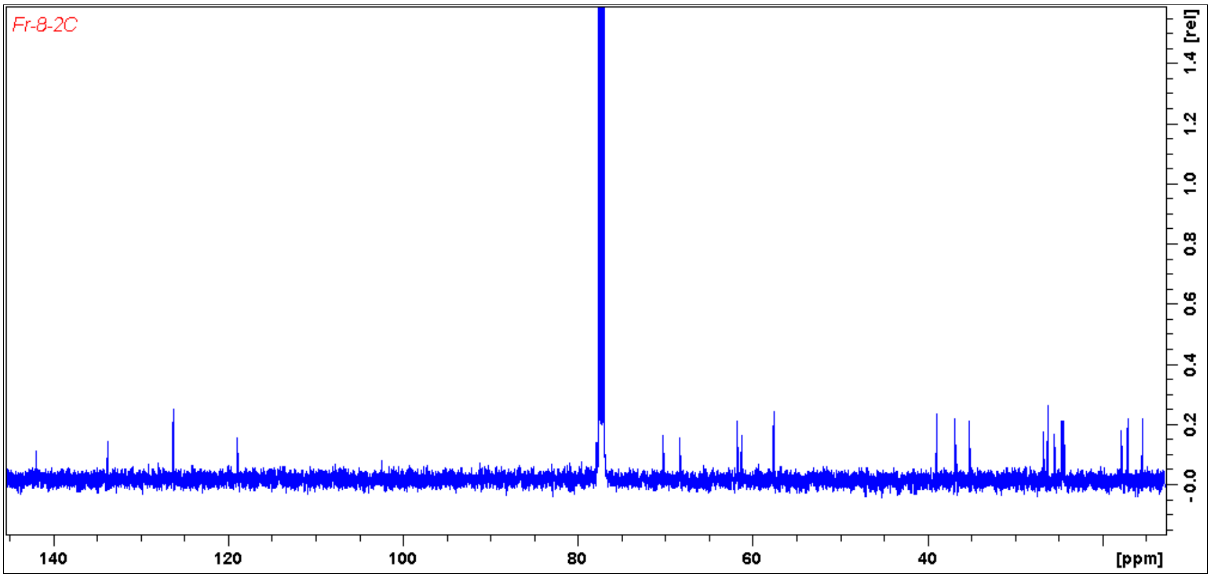

Figure S8. ^{13}C-NMR spectrum (125 MHz) of **2**.

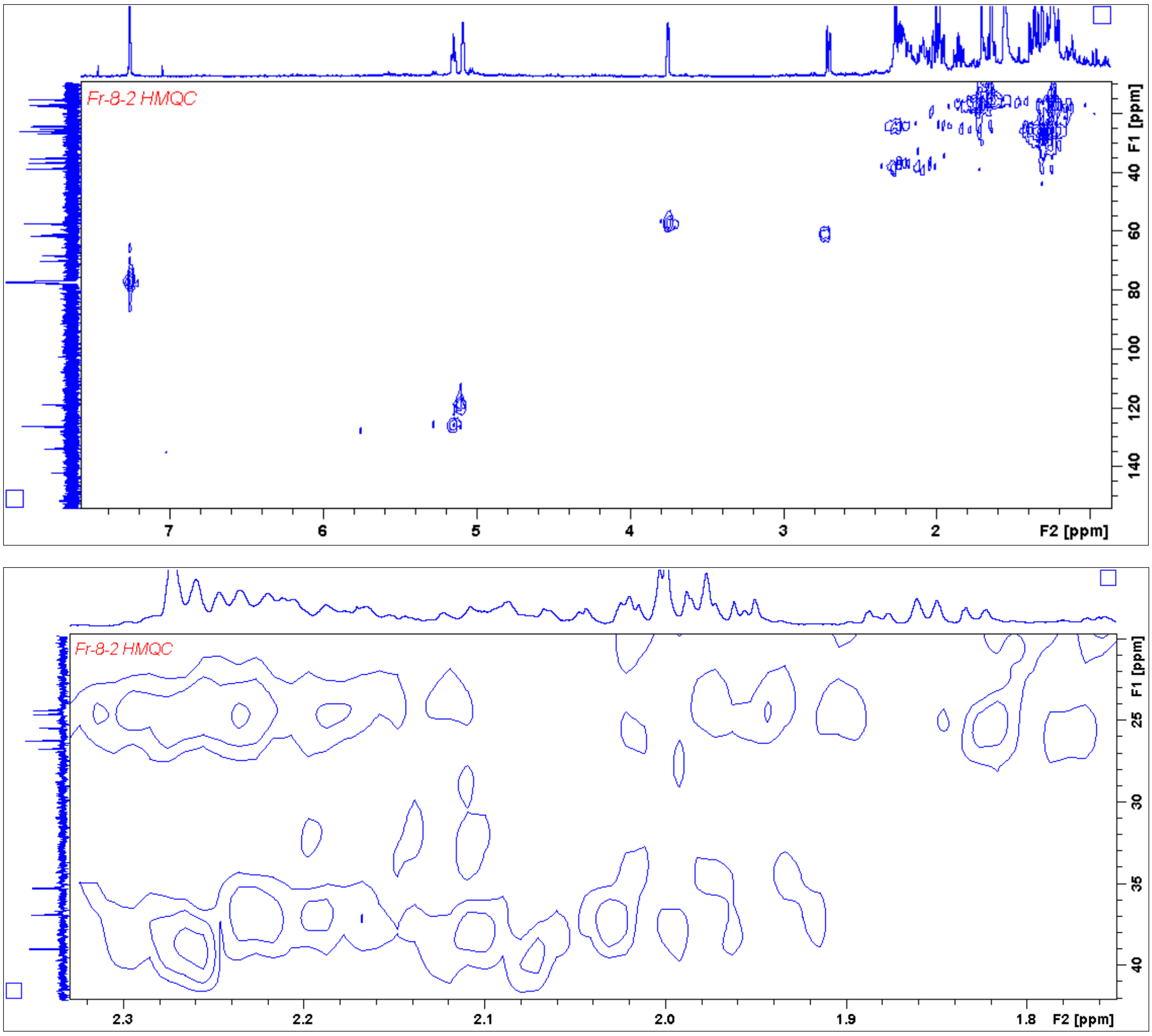

Figure S9. *Cont.*

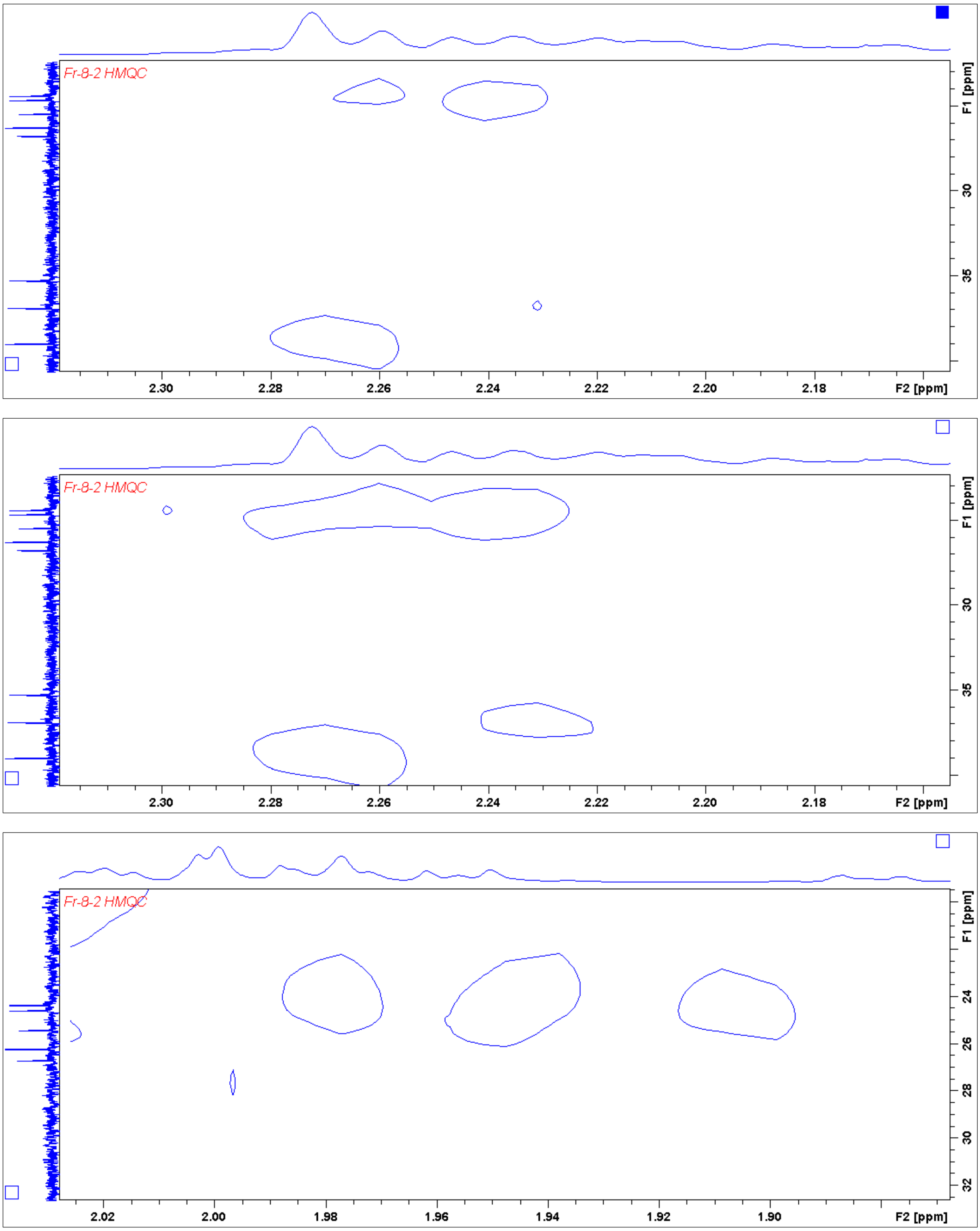

Figure S9. *Cont.*

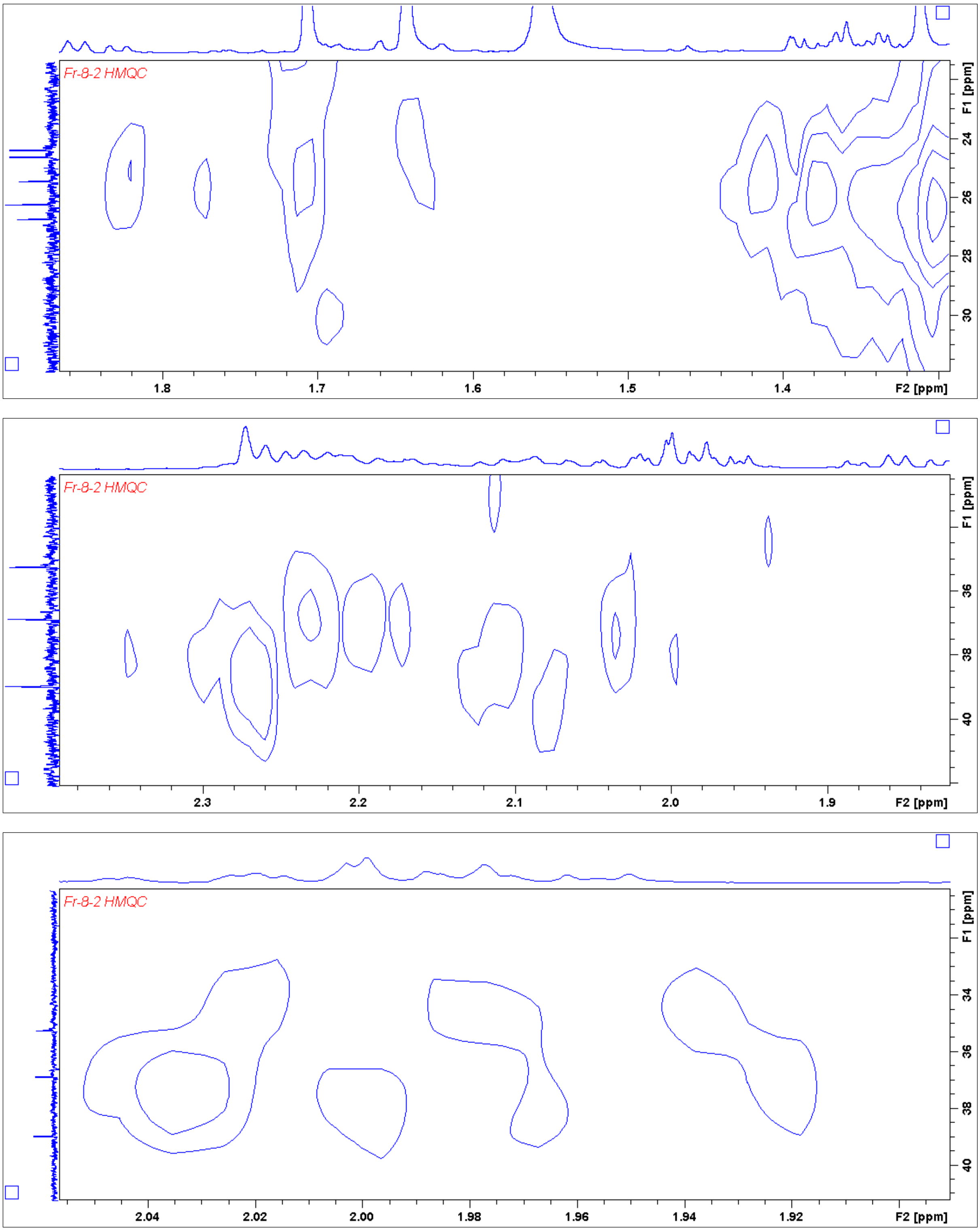

Figure S9. HMQC spectra of **2**.

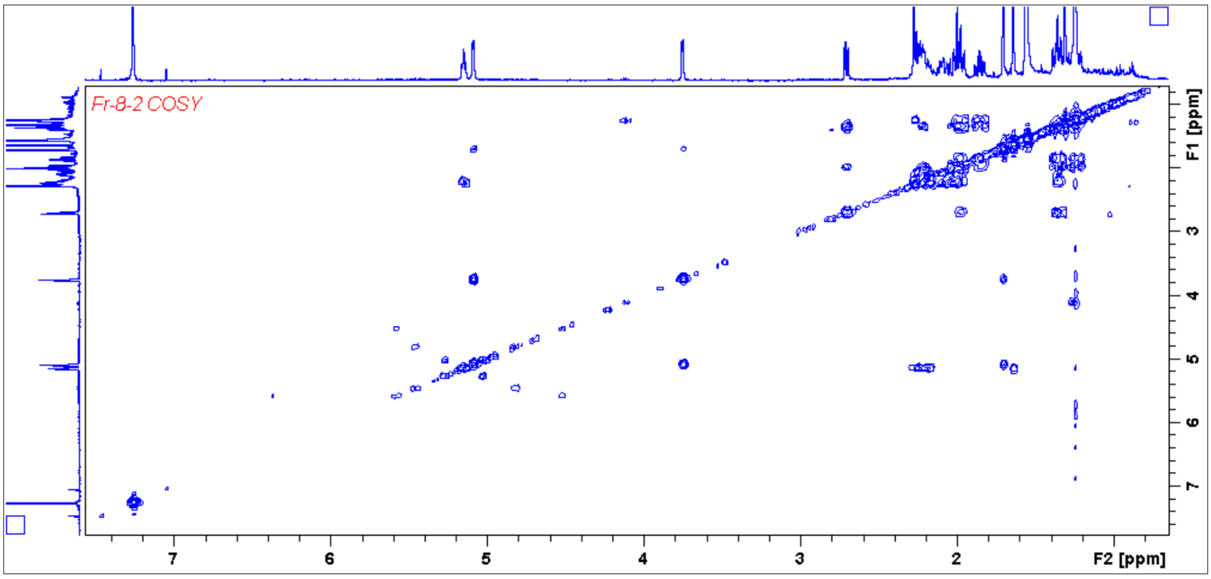

Figure S10. ^{1}H–^{1}H COSY spectrum of **2**.

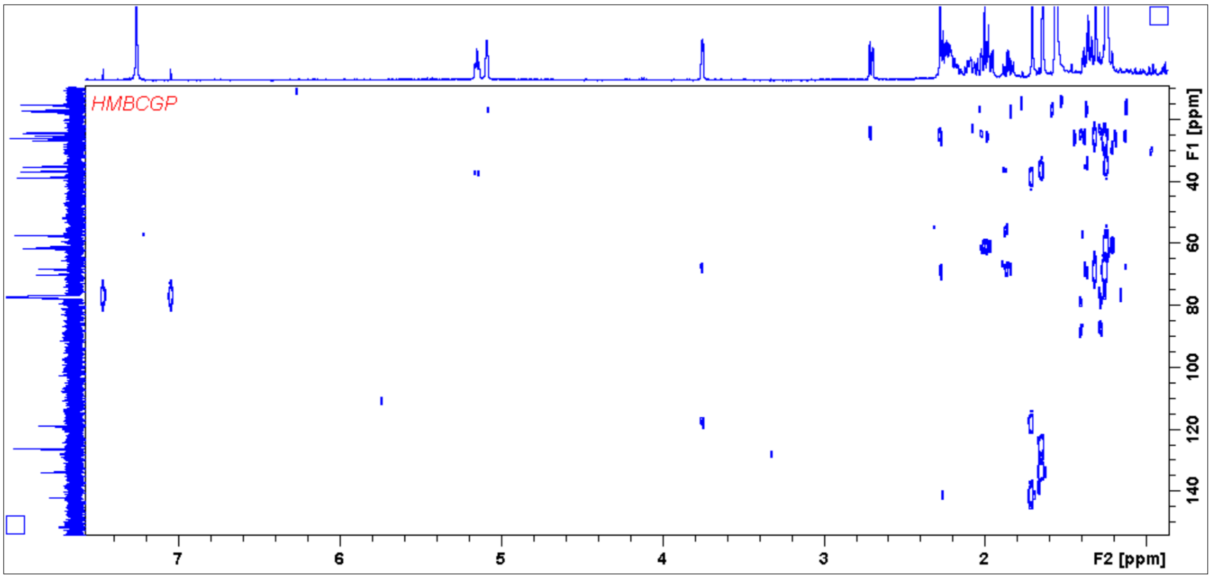

Figure S11. HMBC spectrum of **2** in $CDCl_3$.

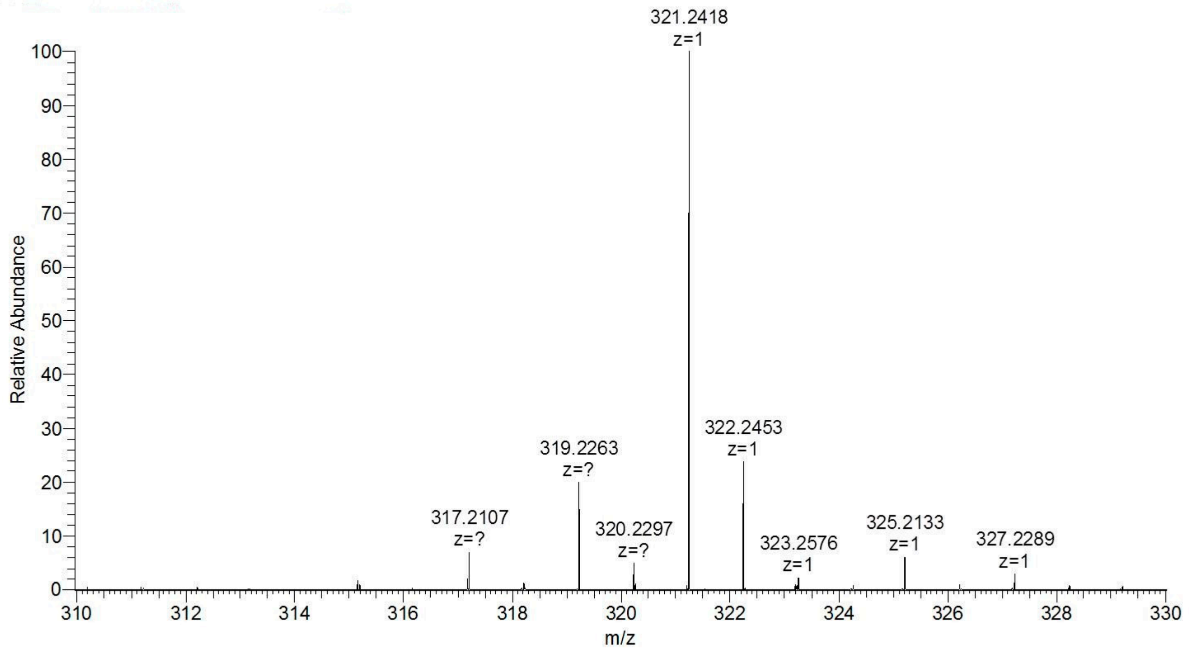

	m/z	Theo. Mass	Delta (mmu)	RDB equiv.	Composition
$[M+H]^+$	321.2418	321.2424	-0.63	4.5	$C_{20}H_{33}O_3$

Figure S12. HRNSIMS spectrum of **2**.

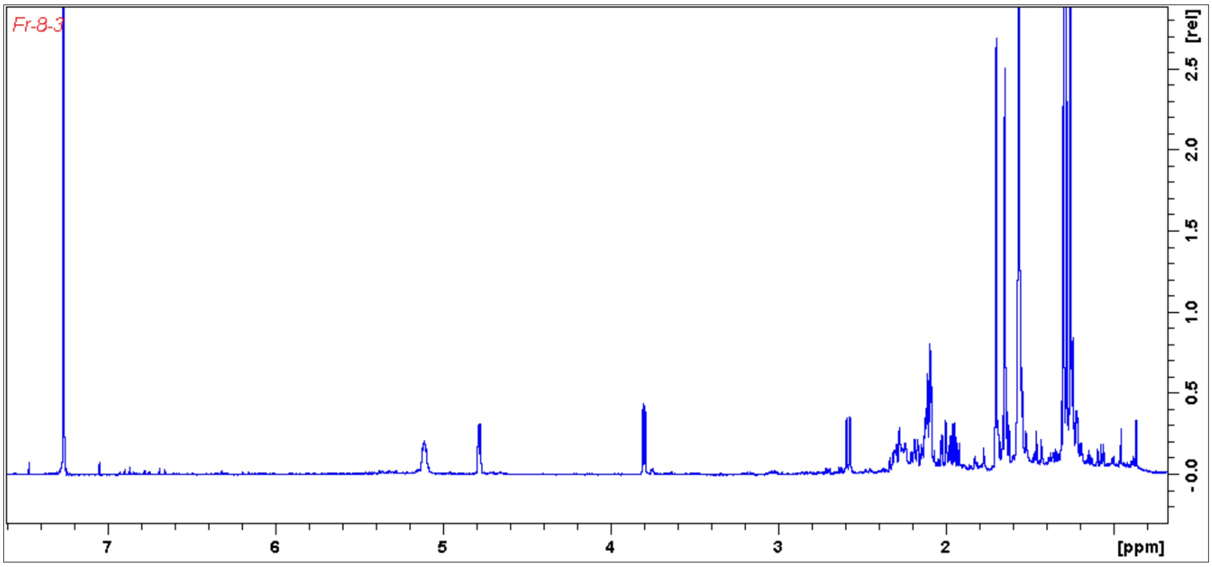

Figure S13. *Cont.*

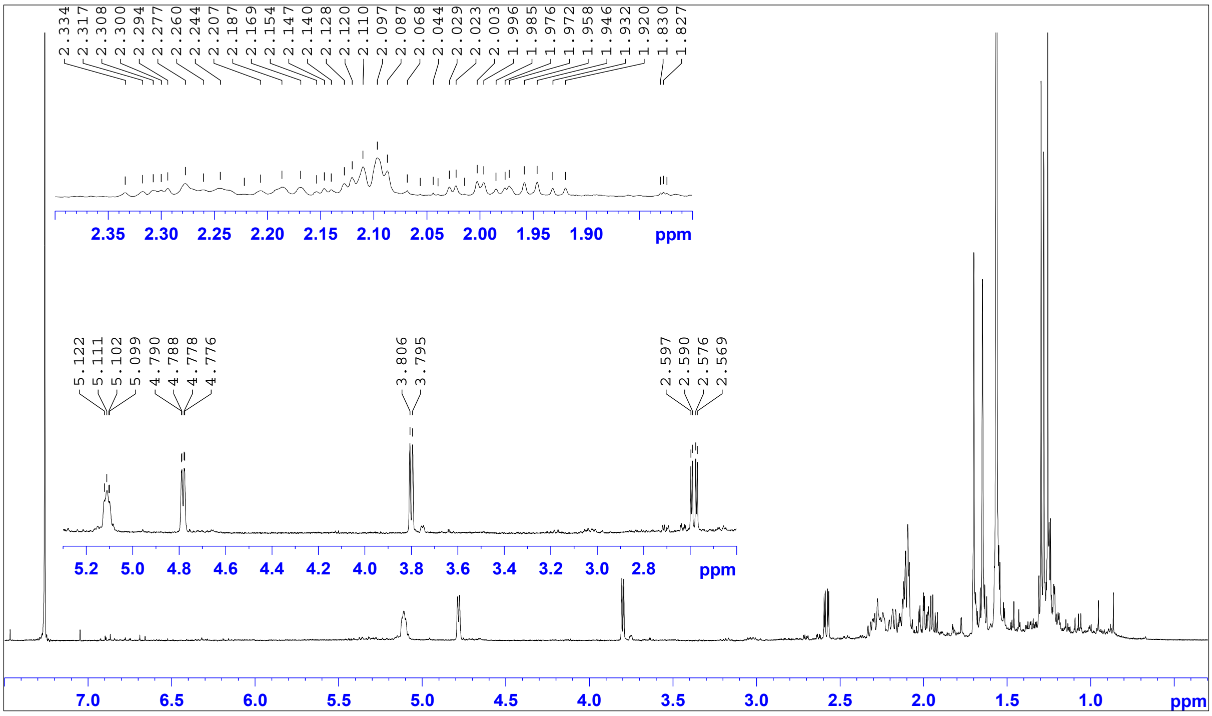

Figure S13. ^{1}H-NMR spectra (500 MHz) of **3**.

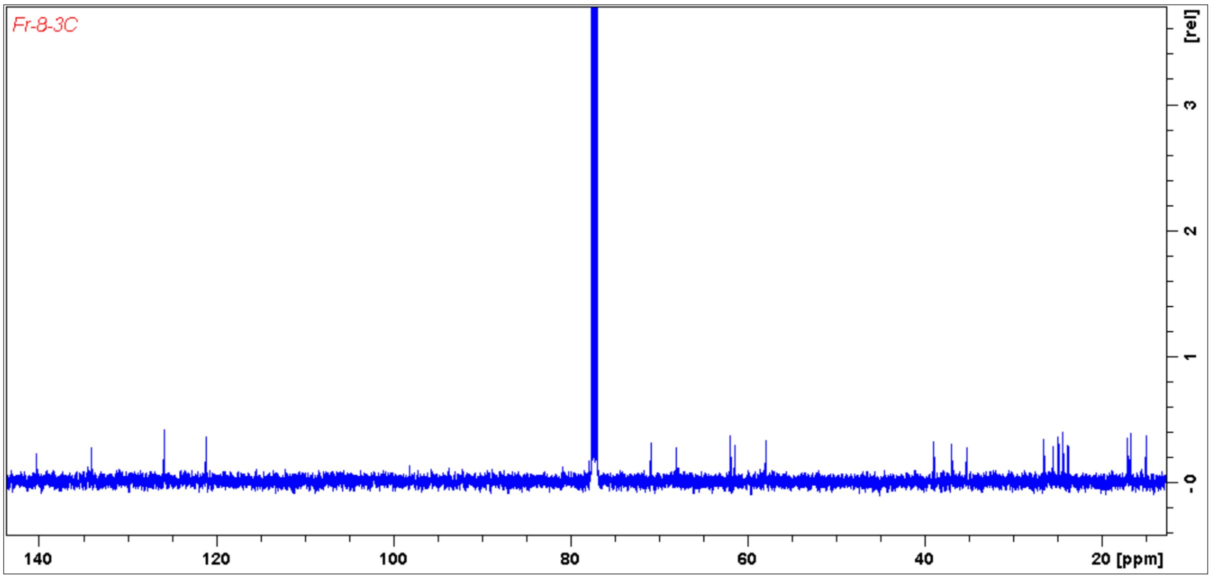

Figure S14. ^{13}C-NMR spectrum (125 MHz) of **3**.

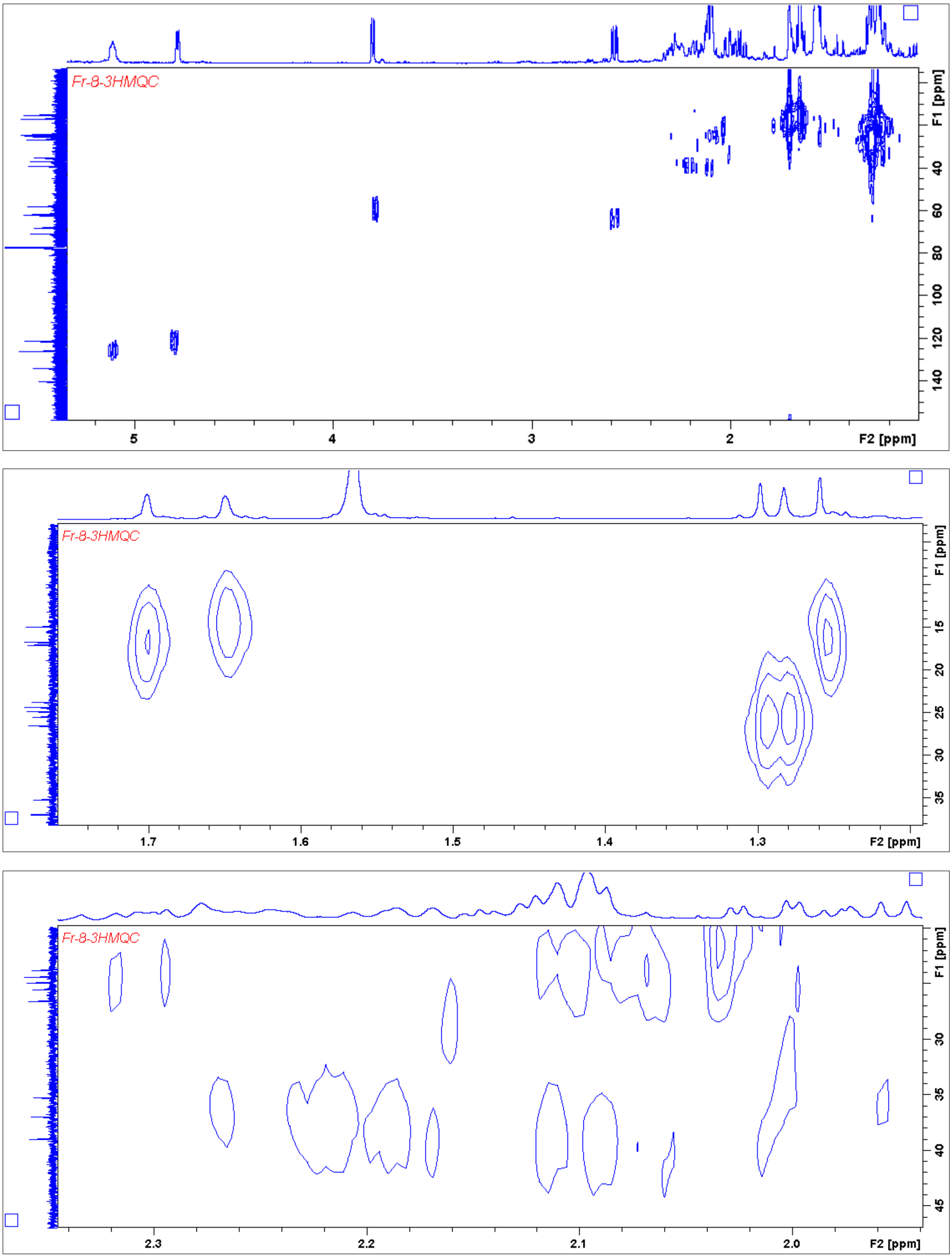

Figure S15. *Cont.*

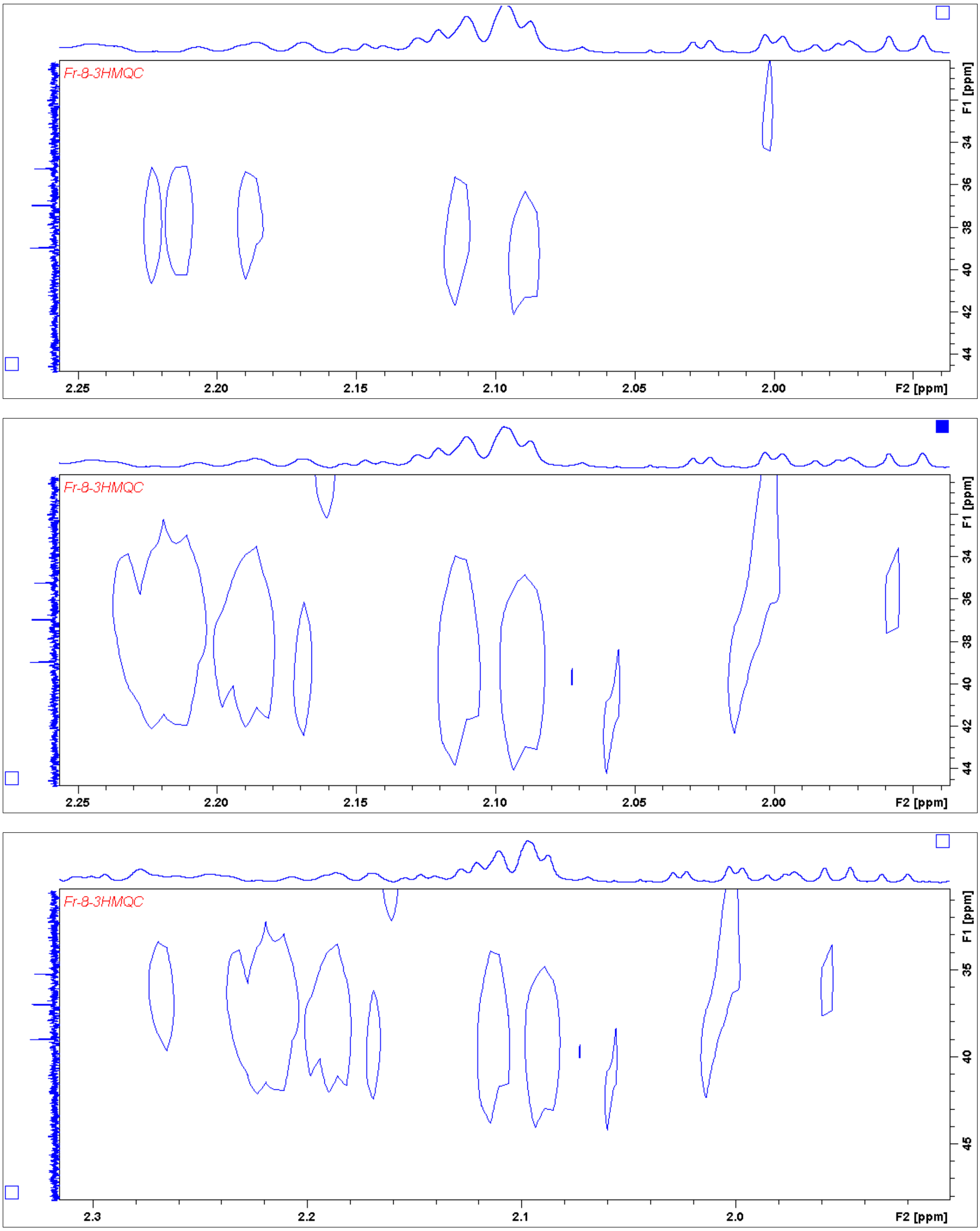

Figure S15. HMQC spectra of **3**.

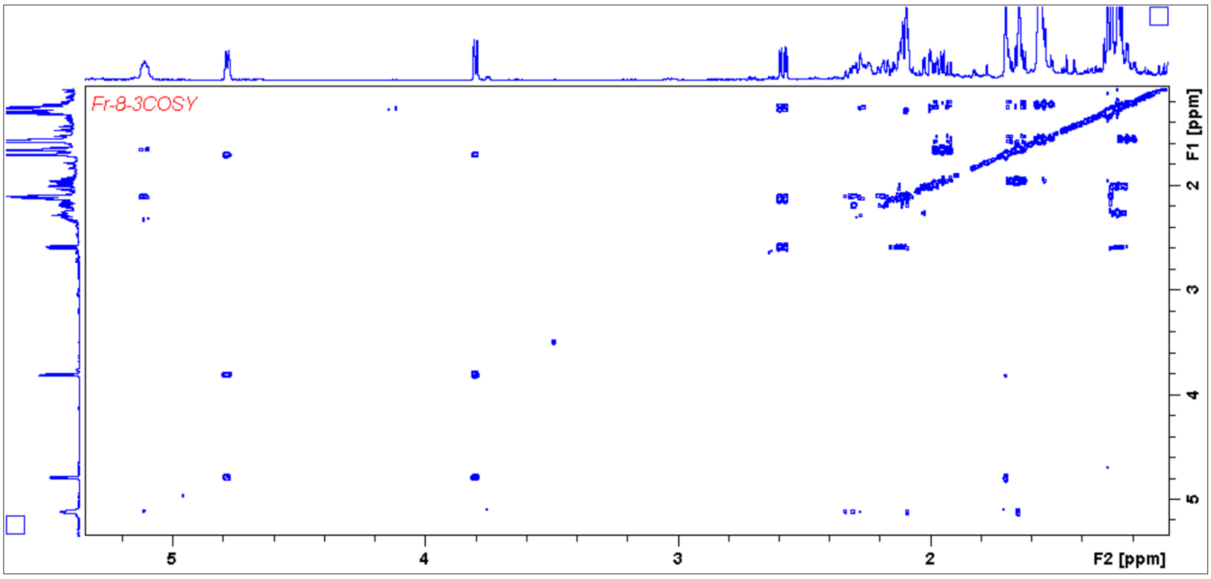

Figure S16. ^{1}H–^{1}H COSY spectrum of **3**.

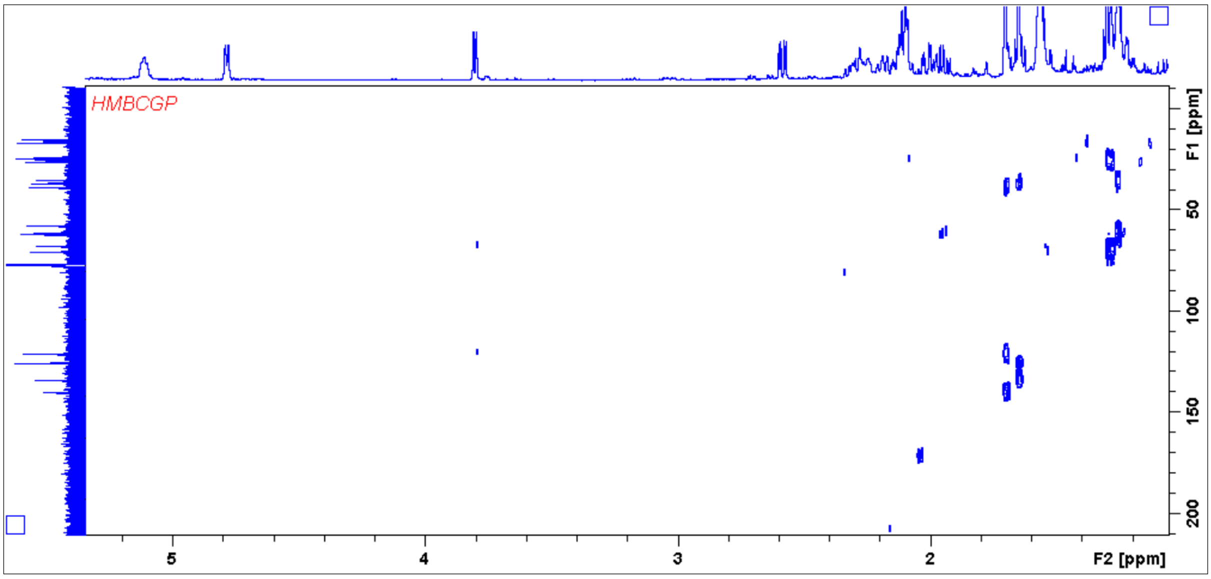

Figure S17. HMBC spectrum of **3** in $CDCl_3$.

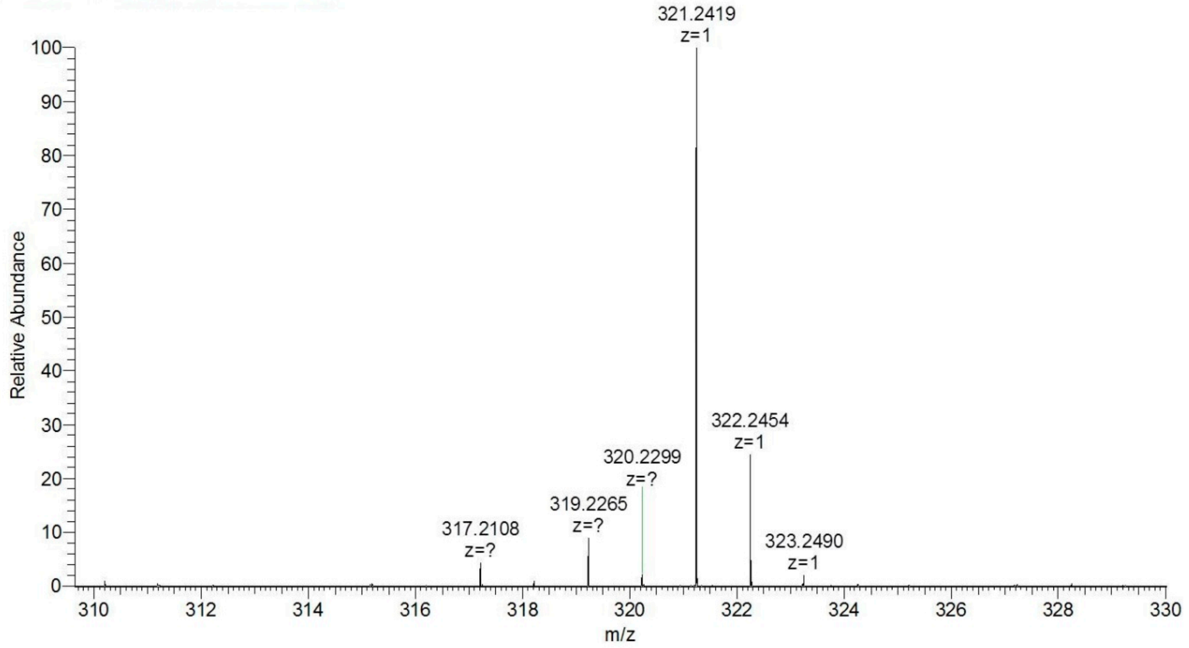

	m/z	Theo. Mass	Delta (mmu)	RDB equiv.	Composition
$[M+H]^+$	321.2419	321.2424	-0.54	4.5	$C_{20}H_{33}O_3$

Figure S18. HRNSIMS spectrum of **3**.
